# Supplementary material for: Long-term cancer survival prediction using multimodal deep learning
Source: Sci Rep. 2021 Jun 29;11:13505. doi: 10.1038/s41598-021-92799-4 (PMC8242026; doi:10.1038/s41598-021-92799-4)
Supplement: Supplementary file 1 — Supplementary Information. [file 41598_2021_92799_MOESM1_ESM.pdf]

Supplementary figures

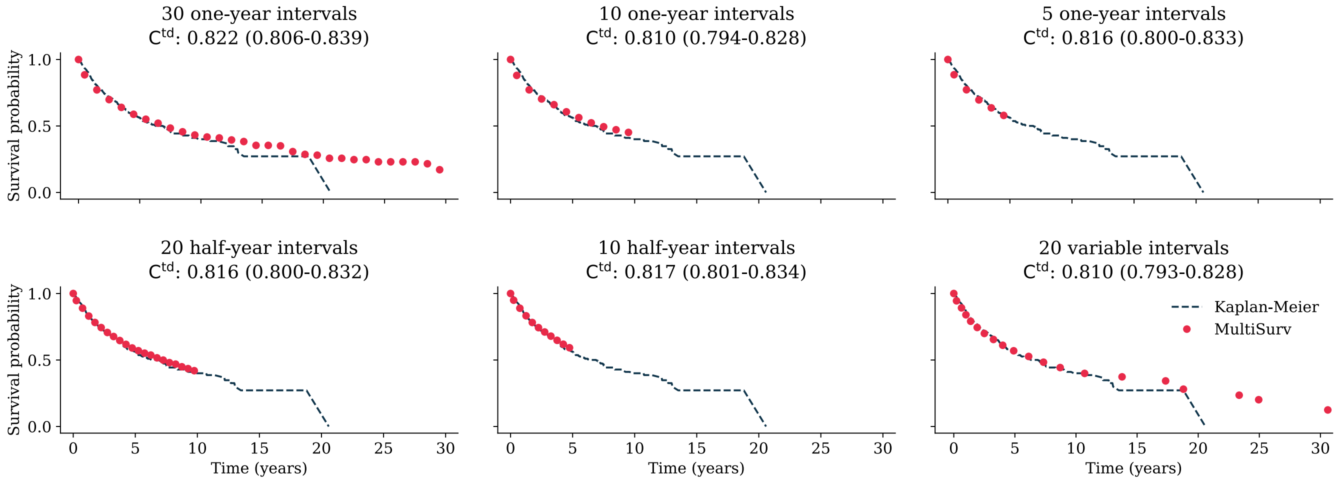

Fig. S1. Survival predictions obtained with MultiSurv models defined with different output intervals. We defined MultiSurv models with different output time intervals and trained them on clinical and mRNA data using the default settings described in the methods section. We chose five equidistant interval schemes: 30, 10, and 5 yearly intervals; 20, and 10 half-year intervals. Additionally, we tested a grid of 20 variable-length time intervals defined by quantiles of event times in the training data, as determined by constant decreases in the Kaplan-Meier survival estimates [1]. Each panel shows the mean of the respective model's prediction for all test patients at each each time interval. To facilitate comparison, the predictions are overlayed on the Kaplan-Meier estimation. Panel titles indicate the model's output time intervals and the time-dependent C-index (C^{td} with 95% confidence intervals). It can be seen that all models yield predictions with comparable C^{td} and similar to the Kaplan-Meier estimate.

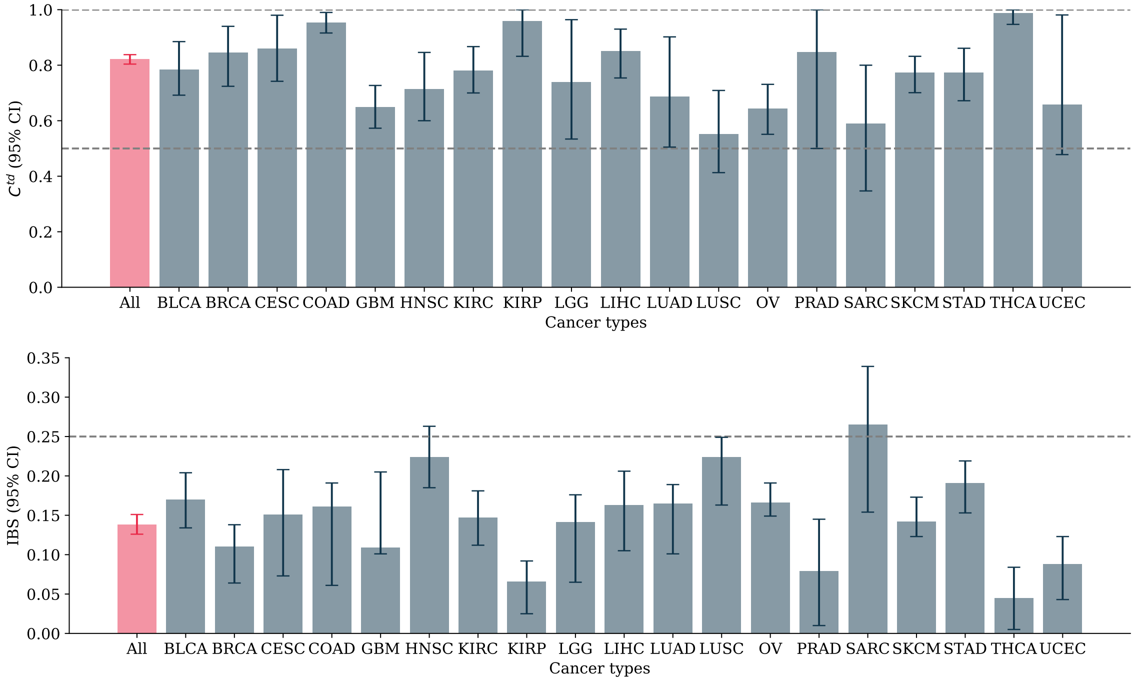

Fig. S2. MultiSurv performance by cancer type. Pan-cancer and individual cancer type time-dependent C-index (C^{td}) and integrated Brier Score (IBS). Cancer types with less than 20 patients in the test data were excluded to avoid noisy values.

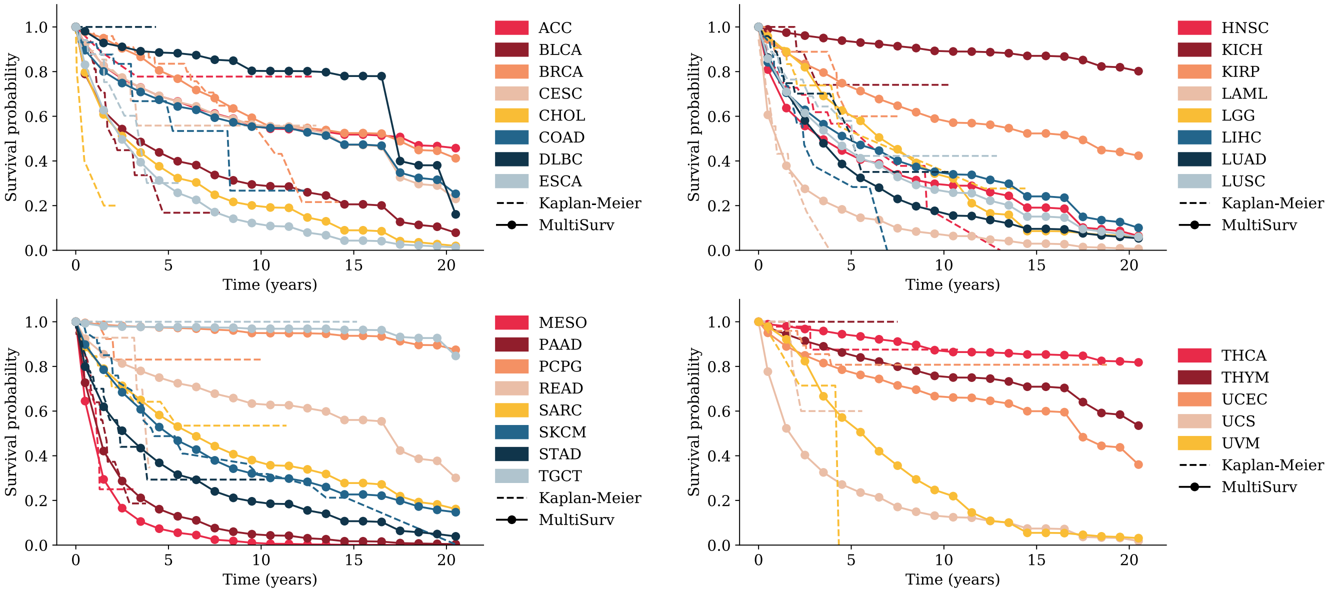

Fig. S3. MultiSurv predictions for individual cancer types. Cancer types (all except those already included in panel c) of Fig. 2) are split between the four panels for clarity. Data points are mean values of patient survival predictions for each discrete-time output interval, overlayed on the corresponding Kaplan-Meier estimations.

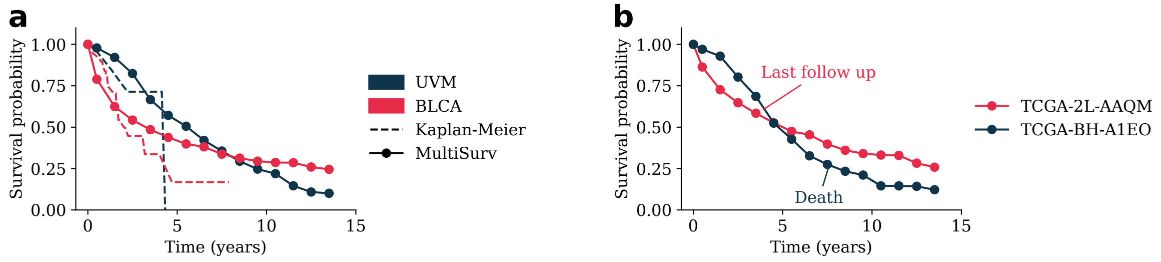

Fig. S4. Violation of proportional hazards assumption. a) Survival curves constructed from Kaplan-Meier estimator outputs for two example cancer types, Uveal Melanoma (UVM) and Bladder Urothelial Carcinoma (BLCA), with UVM showing better short-term prognosis, within the first five years, turning to worse prognosis in the long term. MultiSurv prediction survival curves averaged for all patients diagnosed with each of the two cancer types reproduce the proportional hazard violation, even if the curve intersection point occurs later (around year eight). **b)** MultiSurv prediction survival curves for two example patients illustrating a similar violation of proportional hazards. Patient TCGA-2L-AAQM is a 52 year-old male diagnosed with Pancreatic Adenocarcinoma (PAAD) who was alive at last follow up 1,383 days after diagnosis; TCGA-BH-A1EO is a 68 year-old female diagnosed with Breast Invasive Carcinoma (BRCA) who died 2,798 days after diagnosis. This prediction example seems sensible, considering cancer type prognosis and patient age (without accounting for additional patient features). Early prognosis ranking of the two patients is well aligned with overall cancer type prognosis (PAAD's prognosis is worse than BRCA's). The BRCA patient is 16 years older than the PAAD patient, so it is reasonable to expect the former patient's prognosis to become poorer in the long term.

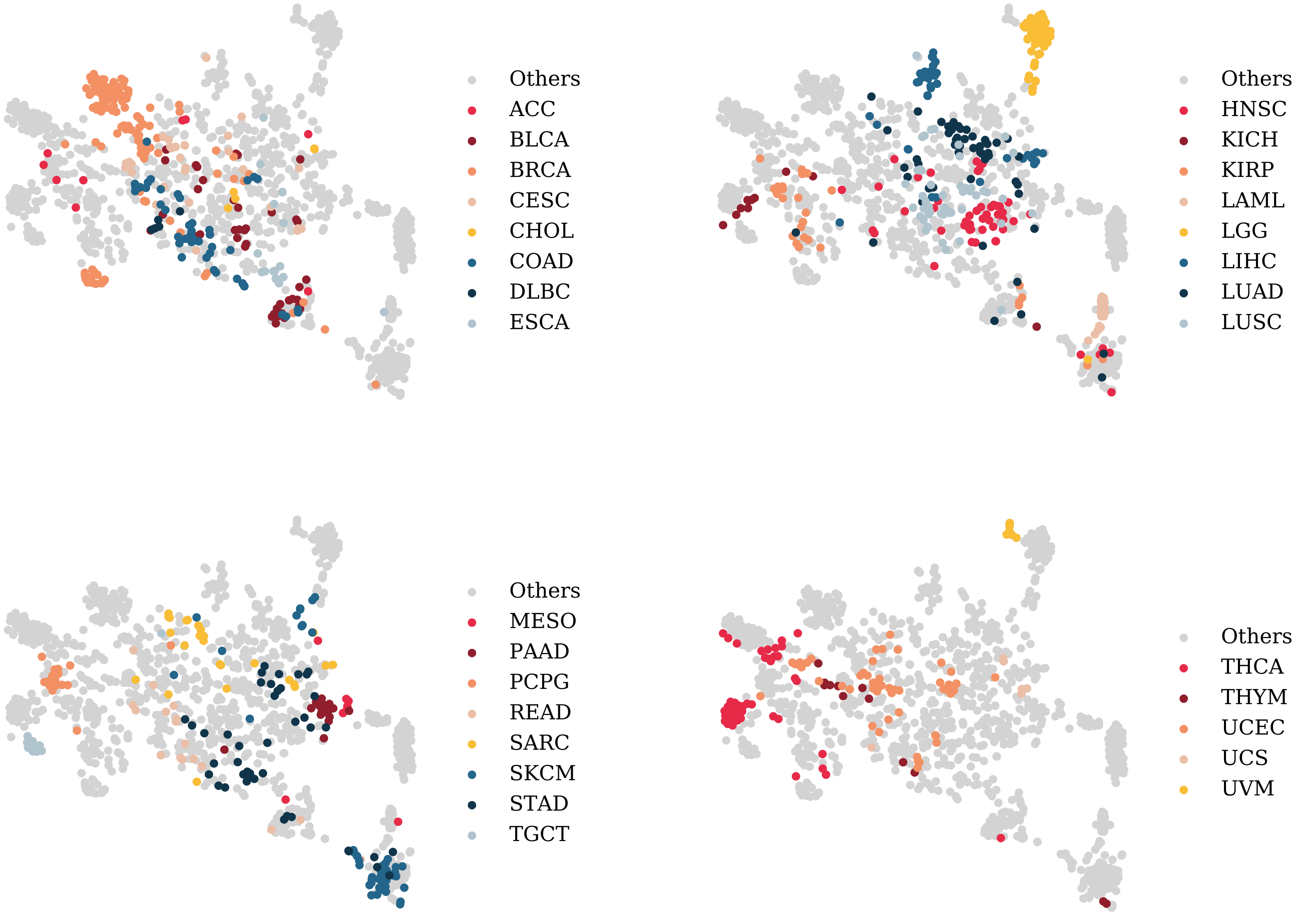

Fig. S5. Visualization of MultiSurv's internal feature represenions for individual cancer types. Two-dimensional t-SNE embedding of the internal fused multimodal feature representation vector of MultiSurv. The MultiSurv model configuration using clinical and mRNA data was used. Cancer types (all except those already included in panel a) of Fig. 3) are split between the four panels for clarity.

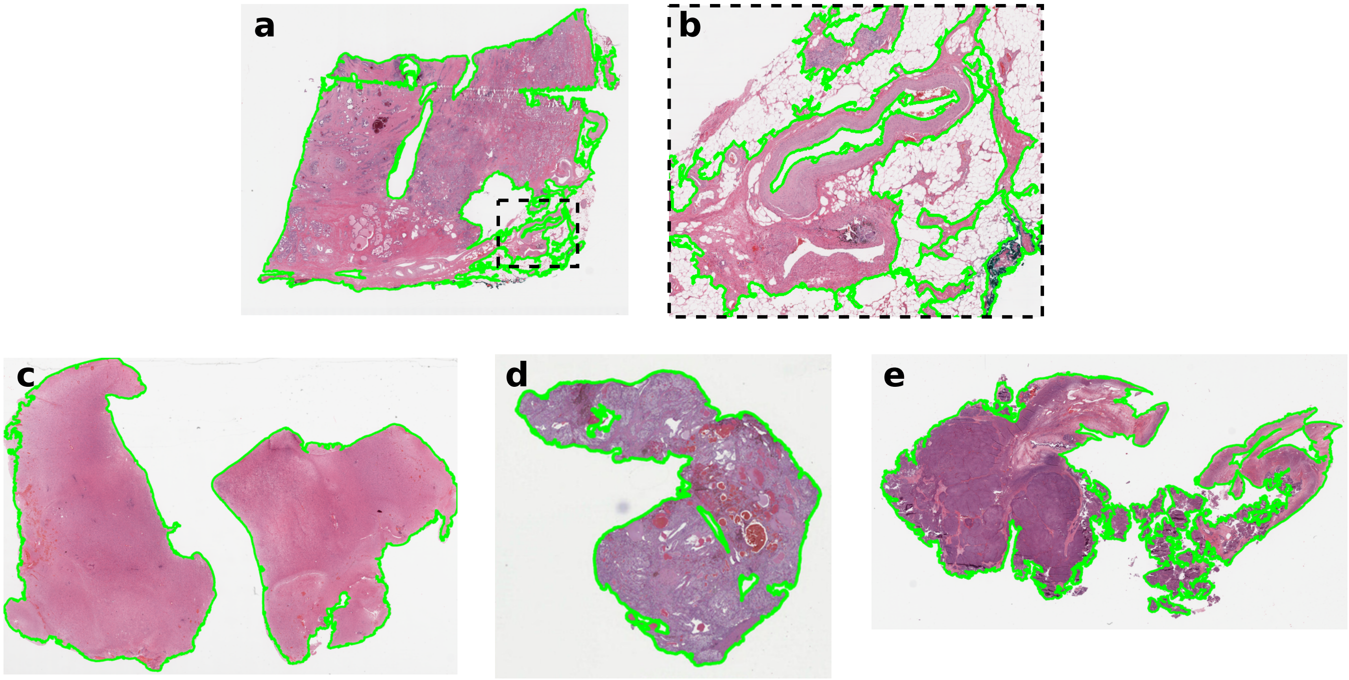

Fig. S6. Example whole-slide images (WSI). The green lines show the borders of the automatically generated binary tissue segmentation mask. **a)** Example WSI from patient diagnosed with prostate adenocarcinoma (PRAD; patient code TCGA-TP-A8TT; slide code TCGA-TP-A8TT-01Z-00-DX1). **b)** Enlarged region delimited by dashed line in a). Example WSI from patient diagnosed with **c)** glioblastoma multiforme (GBM; patient code TCGA-02-0010; slide code TCGA-02-0010-01Z-00-DX3), **d)** kidney renal clear cell carcinoma (KIRC; patient code TCGA-A3-A6NI; slide code TCGA-A3-A6NI-01Z-00-DX1), and **e)** ovarian serous cystadenocarcinoma (OV; patient code TCGA-25-2397; slide code TCGA-25-2397-01Z-00-DX1).

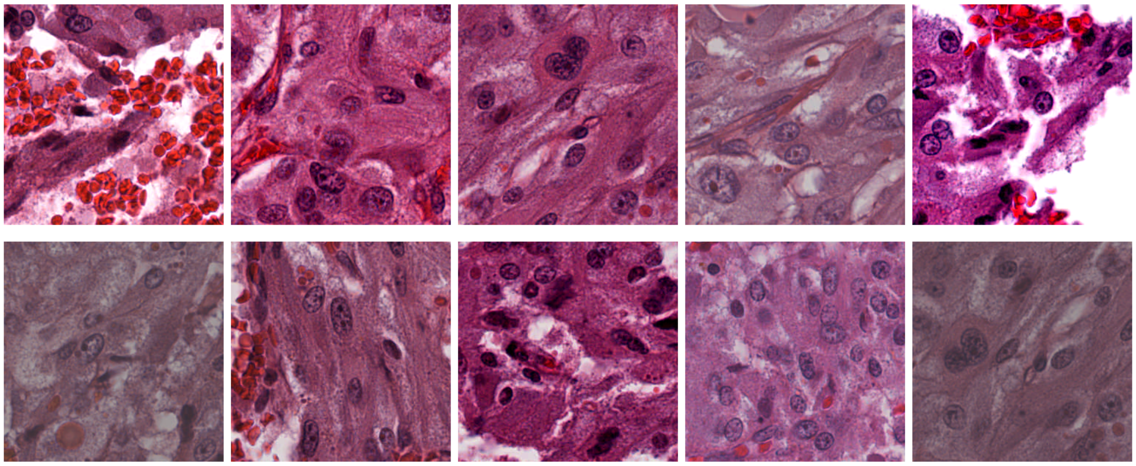

Fig. S7. Example patches sampled from whole-slide images (WSI). Patches of size 299×299 pixels sampled from WSIs from two patients and transformed using the data augmentation procedure used for model training. Top row: patient code TCGA-PE-A5DC, diagnosed with breast invasive carcinoma (BRCA); bottom row: patient code TCGA-DD-AACY, diagnosed with liver hepatocellular carcinoma (LIHC).

Supplementary tables

Table S1. Number of patients and follow up durations for each cancer type.

			Duration (days)		
**Cancer type**	**No. patients**	**% censored**	**Min.**	**Max.**	**Median**
ACC	92	63.0	0	4,673	1,182.5
BLCA	408	55.9	0	5,050	536
BRCA	1,095	86.2	0	8,605	825
CESC	307	76.5	0	6,408	638
CHOL	48	54.2	10	1,976	678.5
COAD	458	77.7	0	4,502	657
DLBC	48	81.2	0	6,425	811.5
ESCA	185	58.4	0	3,714	400
GBM	592	17.1	0	3,881	368.5
HNSC	526	57.6	1	6,417	644.5
KICH	112	89.3	6	5,132	1,471.5
KIRC	537	67.0	0	4,537	1,175
KIRP	290	84.8	0	5,925	767.5
LAML	186	35.5	0	2,861	365
LGG	512	75.6	0	6,423	677.5
LIHC	375	64.8	0	3,675	601
LUAD	513	64.1	0	7,248	656
LUSC	498	56.8	0	5,287	663
MESO	85	14.1	20	2,790	527
OV	582	40.2	8	5,481	1,002
PAAD	185	45.9	0	2741	467
PCPG	179	96.6	2	9,634	755
PRAD	498	98.0	23	5,024	932
READ	169	84.0	0	3,932	609
SARC	261	62.1	0	5,723	938
SKCM	459	51.9	0	11,252	1,124
STAD	434	60.8	0	3,720	424
TGCT	134	97.0	3	7,437	1,261
THCA	507	96.8	0	5,423	944
THYM	123	92.7	14	4,575	1253
UCEC	546	83.3	0	6,859	897.5
UCS	57	38.6	0	4,269	597
UVM	80	71.2	4	2,600	784
All	11,081	67.5	0	11,252	714

Supplementary Note 1: Data preprocessing

We used standard feature selection and feature transformation methods. Clinical and imaging data were handled as described below. Since the majority of features in the high-dimensional omics data modalities are expected to represent little or no predictive value, we used a feature selection procedure to reduce computational cost and facilitate model training. Briefly, we started by ranking features within each modality according to variance over all patients. Then, we performed some limited empirical model training to determine the approximate threshold number of features below which an impact on model validation metrics became noticeable. Finally, features below the determined threshold were dropped. The selected continuous and categorical features were then scaled and encoded, respectively, as described below. The resulting final number of patients and features are listed in Table 3. To fit Cox proportional hazards (CPH) and random survival forest (RSF) baseline models, high-dimensional omics data modalities were further reduced to their 50 principal components returned by the principal component analysis algorithm (PCA; implementation from scikit-learn v0.22.1).

Tabular clinical data. Among the available clinical features, we selected one continuous feature, age at diagnosis, plus nine categorical features: gender, race, cancer type (named "project_id"), tumor stage, prior malignancy, synchronous malignancy, prior treatment, pharmaceutical treatment, and radiation treatment. Five features had missing values for more than 10% of the patients: tumor stage (37.60%), synchronous malignancy (17.21%), pharmaceutical treatment (11.46%), radiation treatment (10.93%), and prior malignancy (10.22%). We replaced missing age at diagnosis values by the feature's median value. We introduced a new categorical level to encode missing categorical feature values. Replacing missing categorical values by the feature's mode instead did not allow any noticeable improvement in results. We then scaled age at diagnosis values to the range between 0 and 1 and encoded categorical feature values as integers between 0 and the number of categories (missing values encoded as a specific category, as mentioned above).

High-throughput omics data modalities. Raw gene expression (mRNA) data consists of upper quartile-normalized fragments per kilobase of transcript per million mapped reads (FPKM-UQ) for 60,483 mRNAs. Micro RNA expression (miRNA) data consists of counts per million mapped reads (RPM) for 1,881 miRNAs. The DNA methylation (DNAm) data includes probe measurements targeting known CpG sites obtained using one of two different technologies: Illumina Infinium Human Methylation 27 and Human Methylation 450. To consolidate the dataset, we used the intersection of probes between the two technologies, resulting in a total of 25'978 Beta values (estimated DNA methylation level at the target CpG site). DNA copy number variation (CNV) data consists of a three-level categorical representation (neutral, gene loss, or gene gain) for a selection of 19,729 protein coding genes. We then transformed the features in these omics data modalities in the same way described for clinical data above: continuous features in mRNA, miRNA, and DNAm were scaled to the range between 0 and 1, while the categorical features in CNV were encoded as the integer values in a count corresponding to the number of categorical levels (starting at 0).

Whole-slide images. Whole-slide images (WSI) consist of digital scans of diagnostic microscopy slides containing biopsy tissue stained with hematoxylin-eosin (H&E). At the highest resolution level, WSIs are gigapixel-level images. In practical terms, using WSIs directly for modeling would require extremely high computer memory and processing resources. In order to avoid this problem, we used smaller image patches instead, sampled from representative regions of interest (ROI) within each WSI. Briefly, we first segmented tissue regions within each WSI. This was done automatically by selecting a downsampled level of the WSI and transferring it from RGB to HSV color space. Then, we generated a binary tissue mask using a threshold value determined in the saturation channel using Otsu's algorithm [2]. Finally, we consolidated the ROI mask by filling in small holes and removing small objects using morphological operations. Example ROIs (i.e. tissue segmentation results) are displayed in Fig. S6. Model input imaging data was then generated by sampling small 299×299-pixel patches from the ROI. During model training, we augmented the data by applying the following transformations to the sampled patches: top-bottom flipping with 50% probability, rotation by a random integer multiple of 90 degrees between 0 and 4, and random color perturbations. The color perturbations consisted of changes to specific image attributes by a factor selected at random from a predefined interval: brightness ($[1-64/255, 1+64/255]$), contrast ($[1-0.5, 1+0.5]$), saturation ($[1-0.25, 1+0.25]$), and hue ($[-0.04, 0.04]$). We performed these transformations using the ColorJitter class in the transforms module of PyTorch's torchvision package v0.5.0. Example transformed tissue patches are displayed in Fig. S7.

Supplementary Note 2: Multimodal fusion layer

MultiSurv's data fusion layer reduces the set of feature representation vectors to a single fusion vector, used as input for the subsequent module. Let $\mathbf{Z} = [\mathbf{z}_1, ..., \mathbf{z}_n]$ be the matrix of feature representation vectors, with $\mathbf{z}_l \in \mathbb{R}^m$ containing the representation of the lth input data modality. The mechanism used in MultiSurv reduces this matrix to a compact fusion vector $\mathbf{c} \in \mathbb{R}^m$ by taking the row-wise maxima, as described in the Methods section. Besides this approach, we tested the alternative schemes described below.

1. Row-wise sum instead of the maximum operation.
2. Row-wise product instead of the maximum operation.
3. Concatenation of the feature representation vectors into a single vector (of length $m \cdot n$).
4. The recently published embracement multimodal fusion layer based on a multinomial sampling mechanism [3].
5. The keyless multimodal attention mechanism previously used for natural video data [4]. This mechanism relies on learned attention weights $\mathbf{A} = [\mathbf{a}_1, ..., \mathbf{a}_n]$, which we computed as originally described or in a modified way by including the hyperbolic tangent (tanh) non-linear function. In this approach, the compact fusion vector $\mathbf{c} \in \mathbb{R}^m$ is obtained as follows:

$$\mathbf{c} = \sum_{l=1}^{n} \mathbf{a}_l \odot \mathbf{z}_l, \tag{S1}$$

where $\mathbf{a}_l \in \mathbb{R}^m$ and $\mathbf{z}_l \in \mathbb{R}^m$ are the attention weights and feature representations, respectively, for the lth data modality and $\odot$ is the element-wise product. For patient i, each attention weight vector is obtained by first computing attention scores $\mathbf{S}^{(i)} = [\mathbf{s}_1^{(i)}, ..., \mathbf{s}_n^{(i)}]$, with each $\mathbf{s}_l^{(i)} \in \mathbb{R}^m$ computed, in our modified version, from the corresponding data modality's feature representation as follows:

$$\mathbf{s}_l^{(i)} = \text{tanh}(\mathbf{W}_l \mathbf{z}_l^{(i)}), \tag{S2}$$

where $\mathbf{W}_l \in \mathbb{R}^{m \times m}$ is the matrix of learned weights for the lth data modality, and then computing the actual attention weights:

$$\mathbf{A}_k^{(i)} = \text{softmax}(\mathbf{S}_k^{(i)}), \tag{S3}$$

where $\mathbf{S}_k^{(i)} \in \mathbb{R}^n$ and $\mathbf{A}_k^{(i)} \in \mathbb{R}^n$ correspond to row k of $\mathbf{S}^{(i)}$ and $\mathbf{A}^{(i)}$, respectively.

In our empirical analysis, these alternatives did not yield consistent improvements over the element-wise maximum operation used in our approach.

References

1. Kvamme, H. & Borgan, Ø. Continuous and Discrete-Time Survival Prediction with Neural Networks. *arXiv:1910.06724 [stat.ML]* (2019).
2. Otsu, N. A Threshold Selection Method from Gray-Level Histograms. *IEEE T. Sys. Man Cyb.* **9,** 62–66 (1979).
3. Choi, J.-H. & Lee, J.-S. EmbraceNet: A Robust Deep Learning Architecture for Multimodal Classification. *Inform. Fusion* **51,** 259–270 (2019).
4. Long, X. *et al. Multimodal Keyless Attention Fusion for Video Classification* in *Proceedings of the Thirty-Second AAAI Conference on Artificial Intelligence 2018* (AAAI Publications, New Orleans, LA, USA, 2018), 7202–7209.
